# Resilience and the role of equids in humanitarian crises

**DOI:** 10.1111/disa.12501

**Published:** 2022-06-20

**Authors:** Cara Clancy, Tamlin Watson, Zoe Raw

**Affiliations:** ^1^ Senior Researcher (Global), The Donkey Sanctuary United Kingdom; ^2^ Head of Research (Global), The Donkey Sanctuary United Kingdom

**Keywords:** climate crisis, disasters, emergencies, equid welfare, humanitarian crises, natural hazards, resilience, SDGs (Sustainable Development Goals), sustainable development, vulnerable communities, working equids

## Abstract

In times of crisis, working equids can play a pivotal role in supporting vulnerable people in lower middle income countries. However, their contributions are rarely acknowledged in academic research, media reporting, international policy, and development initiatives. This paper explores the involvement of working equids in humanitarian emergencies, notably those pertaining to conflict, drought, climate change, and natural hazards. It presents ‘critical cases’, informed by document analysis of policy papers, historical texts, and academic publications. In addition, it includes the findings of semi‐structured interviews with key informants, primarily field staff working for frontline services in crisis zones, conducted in mid 2020. The paper develops evidence on the role of working equids in crisis situations—expanding the concept of ‘resilience’ to include working animals and contributing to recent academic discussions in the fields of disaster and development studies—highlighting their importance for global policy, resilience programming, and disaster risk reduction, including efforts to achieve the Sustainable Development Goals.

## Introduction

Recent investigations demonstrate that working animals can be crucial to people's livelihoods and, as significant economic assets, must be protected from the effects of disasters resulting from natural hazards and complex emergencies (The Brooke, 2016; WHW and TDS, 2020). Working equids are a key part of the social fabric of many lower middle income countries (LMICs): they provide some of the poorest and most marginalised people in the world with an essential source of income and support (Stringer, 2014), the same groups that are vulnerable to shocks and disturbances, with limited capacity to respond (Bankoff, Hilhorst, and Frerks, 2004).

To date, the vast majority of resilience research has overlooked the role of working animals in LMIC communities that are vulnerable to disasters and emergencies.^1^ Working equids can play a critical part in supporting the resilience and recovery of communities that undergo the impacts of humanitarian crises: they can ferry goods and supplies, support rebuilding efforts, and offer a means of transport for people within or without affected areas. They can also help to maintain a sense of cultural identity and community cohesion during times of upheaval. In the context of disasters, working equids can assist their owners in resuming work, restoring income and productivity (van Dijk and Pritchard, 2014; WHW and TDS, 2020). As such, working equids can reduce community vulnerability to the impacts of disasters and emergencies by generating social and economic stability, which is essential for long‐term sustainable development.

Research shows that animal welfare supports human welfare during times of crisis, yet rarely is adequate provision made for working equids in these contexts. We see this as a fundamental oversight given the fact that millions of people around the world rely on working animals for their survival (Rahman and Reed, 2014). The purpose of this paper is to foreground the role of working animals in humanitarian emergencies. In doing so, it seeks to expand the concept of ‘resilience’ to include working animals, which has implications for global policy, resilience programming, and disaster risk reduction, as well as for international development efforts, including work to achieve the Sustainable Development Goals (SDGs) of the United Nations (UN). This work is informed by document analysis, including policy papers, historical texts, and academic publications. In addition, semi‐structured interviews were conducted between June and July 2020 with key informants well versed in the challenges faced by vulnerable communities and their working equids in LMICs during times of crisis. We use primary and secondary data to develop evidence pertaining to the vital role of equids in crisis situations, contributing to recent academic discussions on resilience, disaster studies, and development.

### Resilience, development and humanitarian crises

Over the past 20 years, the concept of resilience has expanded from its roots in ecology (Welsh, 2014) to encompass a range of disciplines, including psychology, geography, and sociology. While there is no universally accepted definition of resilience (Tierney, 2014), most resilience scholars agree that it has to do with the *capacity* to respond, withstand, and/or successfully deal with change—whether at the individual, community, or state level, or in relation to socioecological systems. The Rockefeller Foundation defines resilience as:

*the capacity … of an individual, community or institution to survive, adapt, and grow in the face of acute crises and chronic stresses… . [It is an] activity that requires a multi‐faceted, interdisciplinary strategy and a systems view to grasp … [issues] like chronic poverty and global warming* (Martin‐Breen and Anderies, 2011, p. 2).

For its part, the UN (2009, p. 24) defines resilience as:

*The ability of a system, community or society exposed to hazards to resist, absorb, accommodate to and recover from the effects of a hazard in a timely and efficient manner, including through the preservation and restoration of its essential basic structures and functions.*

These definitions recognise that resilience is both a strategy and a process, whereby people negotiate (and have the capacity to negotiate) the line between stability and change. Resilience characterises, therefore, the ways in which individuals, communities, and/or systems adapt to variability and uncertainty (Brown, 2016). In some cases, original function and identity are maintained despite the ‘shocks’ or disturbances ('bouncing back'); in other instances, structural change is necessary or unavoidable ('bouncing forward'). Resilience is thus a multidimensional concept, pluralistic in its interpretations and meanings (Brown, 2016). Some have described it as a 'slippery concept' (Grove, 2018), whereas others have suggested that it is little more than a ‘buzzword’ (Porter and Davoudi, 2012; Hussein, 2013) and challenged the ‘inherent positivity’ contained within the concept, insofar as resilience is presented as a desirable state or something to strive towards (Brown, 2016, p. 8). According to critics, an overemphasis on the ‘self‐help capacities’ of vulnerable people can overlook the role of politics, power, and agency in structuring social dynamics, neglecting the factors that make some people more vulnerable than others in the first place (Leach, 2008; Cannon and Müller‐Mahn, 2010; Gaillard, 2010; MacKinnon and Derickson, 2012; Fabinyi, Evans, and Foale, 2014). In response, many critical resilience scholars are more interested in how resilience is applied and utilised in different arenas (Brown, 2016; Grove, 2018).

Although resilience is sometimes critiqued as being an abstract concept, this paper takes a more pragmatic approach, adopting a working definition of resilience and the role of equids. For the purposes of this analysis, we are interested in how working animals are positioned within discussions on disaster risk reduction and resilience enhancement in ‘high‐risk’ places and/or among vulnerable communities of LMICs. The concept of resilience has already had a major influence on decision‐making in international development and disaster management (UN, 2005a, 2015a; DFID, 2011). National and international policies are increasingly highlighting the importance of resilience in disaster risk reduction and sustainable development^2^ (UN, 2005b, 2015a, 2015b; UNDP, 2016). Phrases such as ‘resilient communities’, ‘resilient livelihoods’, ‘resilient development’, and ‘climate resilient’ are now common refrains in the international development sector, particularly in light of the recent orientation towards sustainable development (Béné et al., 2014).^3^ Many commentators agree that there is a ‘connection between resilience and sustainability’ (Perrings, 2006, p. 417) and that resilience is a highly relevant concept with respect to sustainable development.

Since the adoption of the SDGs in 2015, there has been an increase in work linking development to the concept of resilience, recognising that shocks and stresses can reverse years of developmental gains and efforts. Concerns about food security, climate change, and global ecological limits are now met with an interest in ‘resilience‐building’ for sustainable development (Brown, 2016). Table 1 illustrates some of the SDG targets that explicitly have an interest in resilience and resilience‐building.

Aid and development agencies, including non‐governmental organisations (NGOs), are increasingly designing projects and interventions based around the idea of resilience (WRI, 2008; DFID, 2011; Christian Aid, 2012; Brown, 2016). Given rising global temperatures and more frequent and extreme climatic events (Seneviratne et al., 2012), millions of poor and marginalised people are likely to suffer the effects of climate change in coming decades. Climate change disproportionately affects these vulnerable communities (Nazrul Islam and Winkel, 2017):

**Table 1:** Sustainable Development Goals and resilience

**Goal**	**2030 target and link to resilience (in bold)**
1. End poverty in all its forms everywhere.	1.5. By 2030, **build the resilience** of the poor and those in vulnerable situations and reduce their exposure and vulnerability to climate‐related extreme events and other economic, social and environmental shocks and disasters.
2. End hunger, achieve food security and improved nutrition and promote sustainable agriculture.	2.4. By 2030, ensure sustainable food production systems and implement **resilient agricultural practices** that increase productivity and production, that help maintain ecosystems, that **strengthen capacity for adaptation** to climate change, extreme weather, drought, flooding and other disasters and that progressively improve land and soil quality.
9. Build resilient infrastructure, promote inclusive and sustainable industrialization and foster innovation.	9.1 Develop quality, reliable, sustainable and **resilient infrastructure**, including regional and transborder infrastructure, to support economic development and human well‐being, with a focus on affordable and equitable access for all.
11. Make cities and human settlements inclusive, safe, resilient and sustainable.	11.C Support least developed countries, including through financial and technical assistance, in building sustainable and **resilient buildings** utilizing local materials.
13. Take urgent action to combat climate change and its impacts.	13.1 **Strengthen resilience** and **adaptive capacity** to climate‐related hazards and natural disasters in all countries 13.B Promote mechanisms for **raising capacity** for effective climate change‐related planning and management in least developed countries and small island developing States, including focusing on women, youth and local and marginalized communities.
14. Conserve and sustainably use the oceans, seas and marine resources for sustainable development.	14.2 By 2020, sustainably manage and protect marine and coastal ecosystems to avoid significant adverse impacts, including by strengthening their resilience, and take action for their restoration in order to achieve healthy and productive oceans.

**Source**: authors, using content available at https://www.un.org/sustainabledevelopment/sustainable-development-goals/ (last accessed on 24 May 2022).

*Climate change forms a substantial threat to the livelihood of smallholder farmers already affected by the increased variability of annual rains* (van Dijk and Pritchard, 2014, p. 51; see also ASFG, 2010).

The emergence of ‘resilience thinking’ in global policy is due to recognition of the need to prepare for and mitigate these effects through coordinated international efforts.^4^ Successful mitigation and adaptation to the consequences of climate change requires an ability to create an ‘enabling environment’ for vulnerable communities, which in turn depends on available resources and the ability to adapt (Klein and Smith, 2003). Evidence suggests that working animals play a pivotal role in creating this enabling environment in LMICs.

### Working equids, resilient communities, and sustainable development

There are an estimated 100 million equids worldwide (FAO, 2011), with 95 per cent believed to be located in LMICs (Starkey and Starkey, 2004), where, as noted, they provide some of the poorest and most marginalised people in the world with an essential source of income and support (Stringer, 2014). Working equids, along with other working animals, support the livelihoods of between 300 and 600 million people globally (Rahman and Reed, 2014). They fulfil a vital role as draught animals in rural areas, ploughing and tilling agricultural land (Starkey and Starkey, 2004), and as pack animals in the transportation of water, building materials, relief supplies, animal feed, and other supplies (Valette, 2015). The use of working donkeys by households in LMICs can contribute up to 100 per cent of annual gross income (see, for example, Admassu and Shiferaw, 2011; Avornyo et al., 2015; Valette, 2015; The Brooke, 2019; Mwasame, 2020), enabling families to access local markets, schools, hospitals, and other essential services. For this reason, several NGOs have identified working equids as key players in global efforts to achieve the SDGs (Valette, 2015; WHW and TDS, 2017, 2020; The Brooke, 2019; Ghislain, 2019).

The UN's Sendai Framework for Disaster Risk Reduction 2015–2030 explicitly recognises that working animals can be crucial to some people's livelihoods and, as significant economic assets, must be protected from the effects of disasters resulting from natural hazards. Priority 3 of the Sendai Framework, investing in disaster risk reduction for resilience, underlines the need ‘to strengthen the protection of livelihoods and productive assets, including livestock, working animals, tools and seeds’ at the national and local level (UN, 2015a, p. 19). Yet, despite their considerable value, working animals (and particularly working equids) are frequently overlooked in international development and resilience initiatives; their welfare is often ignored entirely. As Perry (2017, p. 601) puts it:

*The multiple roles that livestock play in many LMICs have received inadequate attention in poverty reduction strategies and in the international development investment agenda. Equids are not even included in development programmes in these countries or the agendas of national livestock ministries, so their visibility is close to zero.*

The Brooke (2019) similarly finds that working equids are invisible in national and regional livestock planning and policy framing. While there has been increased academic and policy attention to the role and value of livestock, especially cattle, goats, and sheep, in supporting vulnerable communities and/or those in crisis (Lindenmayer, 2008; LEGS Project, 2014, 2018; FAO, 2016; VSF International, 2018), equids are frequently forgotten in relation to international development and humanitarian aid/relief efforts (Pritchard, 2014; Perry, 2017). Mwasame (2020), for instance, notes that the use of and reliance on equids has been consistently neglected in livestock policy documents throughout Africa. This is partly because working horses, donkeys, and mules are generally excluded from the definition of ‘livestock’ (Pritchard, 2014). Only recently were working equids recognised for their contribution to global food security and formally acknowledged in the Committee on World Food Security's definition of ‘livestock’ (CFS, 2016; see also The Brooke, 2016).

We suggest that ideas of resilience, as they appear in the context of international development and disaster relief and recovery, could be enriched by acknowledging working animals as vital social actors in times of crisis. In addition, we suggest that efforts to meet the SDGs could be strengthened by recognising how equids uniquely contribute to the development of more resilient communities.

To clarify our use of terms: we use ‘humanitarian emergency’ in its broadest sense to mean ‘an event or series of events that represents a critical threat to the health, safety, security or wellbeing of a community or other large group of people, usually over a wide area’ (Humanitarian Coalition, 2020). There are different types of humanitarian crisis, including ‘complex emergencies’, such as famine, political insecurity, and violent armed conflict (ReliefWeb, 2008). A ‘disaster’, meanwhile, is a specific type of event that can result in a state of emergency or crisis (WHO, 2020). The UN (2009, p. 9) defines a disaster as: ‘A serious disruption of the functioning of a community or a society involving widespread human, material, economic or environmental losses and impacts, which exceeds the ability of the affected community or society to cope using its own resources’. Thus, disasters are characterised by impacts that overwhelm the capacities of local responders (UN, 2020). They can be sudden events, such as earthquakes or hurricanes, or slow‐onset events, such as prolonged drought. It is widely recognised that disasters are complex and often social as well as natural. The expression ‘natural disaster’ is purposefully omitted from official UN documents since it ‘conveys the mistaken assumption that disasters occurring as a result of natural hazards are wholly “natural”, and therefore inevitable and outside human control’ (ReliefWeb, 2008) Instead, disasters are the product of the way in which individuals, societies, and political systems relate to threats originating from natural hazards, before and after they have occurred. In the words of Claus et al. (2015, p. 291):

*… rather than events in isolation, disasters are an accumulation of specific economic, political, and social histories* (see also Bryant, 2015).

Social processes generate unequal exposure to risk and so the ‘impacts’ of disasters are always uneven; stratified along lines of class, race, gender, and socioeconomic standing (Kimura, 2015). It is often poor and marginalised populations that suffer the most in the event of a disaster due to a lack of adequate housing, poor infrastructure, limited access to health services, and the challenges brought by being in a low income, financially vulnerable household (Nazrul Islam and Winkel, 2017). These factors mean that not only is there immediate human suffering in the wake of a disaster, but also people from low income households will frequently struggle to rebuild their lives and livelihoods (He et al., 2018). In this paper, we explore the role(s) of working equids in supporting vulnerable communities in LMICs affected by disasters, including how they can help their owners to resume work, restore income, and reinstate livelihoods and productivity (van Dijk and Pritchard, 2014; WHW and TDS, 2020).

## Methods and materials

We reviewed academic and grey literature, including peer‐reviewed articles, institutional reports, working papers, government documents, and media accounts. Outputs were identified using a snowball technique (Echeverri et al., 2018), whereby key search phrases were pinpointed and investigated.^5^ Each was reviewed for relevance, based on the extent to which it discussed and/or provided new information on the role of equids in humanitarian crises. Reference lists were checked to find further relevant pieces of work, which were then reviewed. This process was repeated until no new returns were received (Biernacki and Waldorf, 1981). In addition, evidence was gathered via personal communications with United Kingdom‐based humanitarian and veterinarian organisations, particularly those with overseas field operations. Where possible, we conducted semi‐structured interviews, between June and July 2020, with key informants, primarily field staff of frontline services in crisis zones. Together, this yielded the evidence‐base for a collection of ‘critical cases’ (Yin, 2013) —selected because (i) they have received little (or no) attention in the academic literature to date, and (ii) each uniquely demonstrates the critical (yet varied) role of equids in humanitarian crises. The case studies are organised according to the role of working equids in different types of disasters and emergencies: equids and natural hazards; equids and armed conflicts; equids and climate‐related crises; and equids and humanitarian aid.

## Case study evidence

### Equids and natural hazards: providing relief and recovery after the 2015 earthquake in Nepal

Disasters are complex and often social as well as ‘natural’ (Claus et al., 2015; WHO, 2020). This was demonstrated on 25 April 2015, when Nepal experienced one of the worst earthquakes on record since the Bihar–Nepal earthquake on 15 January 1934. Nearly 9,000 people died and a further 22,000 were injured; entire villages were flattened; and sacred monuments were destroyed (UNESCO, 2015). Poor households suffered the most, with people working and residing in low‐cost and informal buildings that have a high risk of failure (Cross, 2015). Experts found that poor urban planning amplified the fatal force of the disaster:

*Unplanned urban development in the Kathmandu Valley has led to rapid and uncontrolled sprawl; irregular, substandard, and inaccessible housing development; loss of open space, and decreased liveability. It has also increased vulnerability to disasters, making Kathmandu one of the most earthquake‐vulnerable cities in the world* (Muzzini and Aparicio, 2013, p. 7).

Disasters like the 2015 earthquake in Nepal serve as a reminder that pre‐disaster planning and adequate investment in local infrastructure and services are a fundamental part of disaster resilience, supporting communities that may suffer the most. The earthquake struck the central hills and mountains of Nepal; the five worst affected districts/municipalities (in terms of population size and number of people impacted) were: Dhading; Gorkha; Sindhupalchok; Ramechhap; and Nuwakot (OSOCC, 2015). Communities in the most inaccessible regions received initial emergency relief via helicopter, although the availability of only two helicopters throughout the country (OSOCC, 2015, p. 6) meant that alternative land‐based distribution methods had to be relied upon during the early and long‐term recovery phases in mountainous districts, once tracks had been sufficiently repaired for access on foot. One such distribution method was the use of mules, which are a hybrid equid resulting from breeding between a horse and a donkey.

Working mules can be an essential part of rural life in many remote communities where motorised vehicle access is limited or non‐existent (Arriaga‐Jordan et al., 2005; TDS and Animal Nepal, 2016). This reliance becomes more pressing when, during earthquake recovery, working equids become the primary means of aid distribution in these areas, alongside human porters carrying some lighter items. Equids are agile, able to traverse difficult terrain when road access by motorised vehicle becomes impossible; they are also potentially easier to sustain than motor vehicles when fuel and spares supplies are likely disrupted. In Gorkha, for example, mules carried blankets, foodstuffs, and building materials, such as sand, cement, and the wire needed to construct gabions to strengthen embankments once recovery efforts began after the initial emergency response.^6^ These supplies gave people sustenance and warmth, enabled the rebuilding of homes, schools, businesses, and infrastructure, and, in so doing, established an essential link with the reconstruction of isolated rural communities. Since the mule owners hired during recovery efforts were those already working in local communities, their employment supported continued livelihood generation, which is an essential part of the recovery process in isolated regions (LEGS Project, 2014).^7^ Working mules thus have the potential to contribute to the long‐term resilience of poor, remote mountain communities that are vulnerable to the effects of disasters such as the 2015 earthquake in Nepal.

### Equids and armed conflicts: supporting refugees and internally displaced persons

Media reports pay much attention to people who are displaced by conflict, whether internally within their own country or across a border as refugees. Yet, what is little known and rarely documented in media reports is the vital role of working animals in these complex emergency contexts. Refugee and IDP (internally displaced person) encampments offer an interesting example of the shared vulnerability and/or resilience of humans and nonhuman animals. Camps are risky spaces: for instance, hygiene and biosecurity may be poor and so they can be particularly vulnerable to the outbreak and spread of disease among both humans and animals (Beirne and Kelty‐Huber, 2015; Angeloni and Carr, 2018; Owczarczak‐Garstecka, 2018).

Refugees will often bring their animals to camps during emergencies (Alshawawreh, 2018). According to Owczarczak‐Garstecka (2018, p. 11), 'animals that flee with their owners may be exposed to new diseases to which they have no immunity' or, conversely, ‘they may themselves carry diseases to which local animal populations are susceptible’. By way of example, since 2011, Syrian refugees fleeing into Lebanon have been accompanied by thousands of unhealthy, unvaccinated goats, sheep, and cattle, which has posed a biosecurity risk to the local population (Beirne and Kelty‐Huber, 2015). For this reason, says Alshawawreh (2018, p. 8), ‘camp planners and managers need to take animals’ needs into greater account'. Nevertheless, the risks and benefits of human–animal interactions in camp settings are rarely investigated by camp planners and policymakers.

In recent years, scholars have sought to emphasise the importance of animals in the lives of displaced people, including those in encampments (Herz, 2012; Rawlence, 2016; Pollock, 2018; White, 2018). While the contribution of animals to the lives of IDPs is normally recognised in economic terms by international agencies,^8^ animals can also contribute to the lives of IDPs and those living in encampments in a multitude of different social and cultural ways (White, 2020) that support their resettlement elsewhere. People displaced from their homes are affected emotionally and psychologically, as well as economically. Animals can provide a reminder of home and a sense of familiarity during these times of upheaval and uncertainty (Alshawawreh, 2018; White, 2020).^9^

Animals can also offer a means of integration into local settlements, contributing to the process of ‘emplacement’, which has both practical and emotional/psychological aspects to it (White, 2020). They can be central to the negotiations that take place between refugees and their host society. For instance, many people who bring their animals to camps need to graze them and this presents opportunities to become familiar with local surroundings and encounter nearby communities. As White (2020, n.p.) puts it, animals can become ‘a means of connection between the people in the camp and the people in the landscape around them’. Through their animals, refugees can begin to trade with their host community, which may provide an entry point into local social affairs, helping overcome cultural differences to yield a common point of reference. That said, animals can also be a source of conflict between refugees and the host community, by creating, inter alia, competition for grazing and pasture (Hoots, 2018). Informants who we interviewed explained that in refugee encampments in Kenya (such as Daadab and Kakuma) and IDP camps in Somalia (such as Kabsa and Qanshale, Dollow and Jeron, and Bulla Arjan in Bulla Hawa), it is highly beneficial for people to own donkeys as opposed to other livestock, since it reduces resource‐based conflicts with host communities. In the words of one interviewee (KI.3):

*Refugees face restrictions mainly due to the lack of work and travel permits, and the complicated process in acquiring these… . When they do acquire work, the wages are meagre, hence owning donkeys is an important source of income as they are paid to ferry goods.*

This demonstrates the importance of equids in helping to embed people within their new communities, including negotiations between refugees and the host society. Integration is an essential aspect of refugee–host relations; in fact, it is a priority area for international advocacy efforts and capacity‐building (UNHCR, 2016). However, rarely do such agencies recognise the centrality of animals in integration processes in LMICs. As this subsection has revealed, working equids can contribute to the practical resettlement of IDPs through the provision of transport and/or income generation. They can also contribute to the process of ‘emplacement’, whereby individuals or communities develop a sense of belonging in their new environments (White, 2020). All of these aspects of resettlement and emplacement are essential to the maintenance of cultural identity and/or community cohesion in times of crisis.

### Equids and climate‐related crises: drought in the Horn of Africa

In climate‐affected areas, particularly those that experience recurrent and/or prolonged drought, community livelihoods are especially vulnerable and climate‐induced ‘shocks’ can have catastrophic consequences for health and food security (Asiimwe, Ainembabazi, and Egeru, 2020). As global temperatures rise, arid and semi‐arid lands are likely to increase in number, putting further pressure on ecological and agricultural systems already under strain, and leaving them even more vulnerable to climate‐related events (Field et al., 2014). Livestock is often the main source of income for many of those living in Africa's drylands (WFP, 2019; FAO, 2016).

When drought affects these already water‐scarce areas, communities can lose large numbers of livestock owing to the lack of available fodder (VSF International, 2018). In response, some communities in East Africa are diversifying their livestock to include more drought‐resistant species, such as camels and donkeys (Watson, Kochore, and Dabasso, 2016; Asiimwe, Ainembabazi, and Egeru, 2020). Donkeys are known for their ability to survive in areas with sparse vegetation and little water (Klingel, 1990). Like other asses, donkeys have evolved to survive on low‐quality diets, utilising tree roots, bark, and other dry fibrous matter (Izraely et al., 1989). They expend less energy on foraging as compared to cattle, which means that they can spend more time travelling in search of food, accessing remote sources of forage that are inaccessible to cattle (Smith and Pearson, 2005). Consequently, during times of drought, donkeys may have a survival advantage over other animals, due to their ability to tolerate thirst, rehydrate rapidly, and extract necessary nutrients from limited supplies of low‐quality forage (Smith and Pearson, 2005). A frontline field officer based in northern Kenya (KI.7) explained that donkeys are essential in times of drought:

*Donkeys are used to ferry water and food from aid/NGO distribution points to distant villages and herders in remote areas that are experiencing drought, where water boreholes have dried up …. The cattle get very weak during droughts, so the donkeys are used to transport feed to them… and on their journey, the donkeys will scavenge for vegetation like bark and roots and so on… If water and food is scarce, donkey owners will sometimes sprinkle salty water on carton boxes, which reduces the burden of maintenance.*

In 2016, donkeys were used to distribute hay and ranch cubes (concentrated feed) to rural settlements in Korbesa and Dadacha Bassa in Isiolu County, Kenya; bags of feed weighing 50 kilograms per item were delivered to more than 200 households in each settlement. So not only are donkeys important in providing transport of food for people, but they also enable the survival of other livestock species during times of drought. A veterinarian who had worked for many years in drought‐affected areas in South Sudan and Ethiopia (KI.2) observed that donkeys were often the last animal to die in a drought:

*In 2016/2017, there was a very severe drought in South Omo [Ethiopia] and many, many animals were lost … [but] the donkey would be the last to go. People would say, ‘if you see the donkey dying then you know, soon, the people are dying’.*

By keeping donkeys, families enhance household resilience to ‘shocks’ such as droughts. The veterinarian (KI.2) explained that because donkeys tend to survive, they are a lifeline for families after a drought: ‘time‐saving, labour‐saving, and income‐generating; allowing people to rebuild their lives after a drought’. He added that in chronic emergency situations, such as those wrought by drought and famine, the problem of food insecurity is paramount:

*If you have a donkey, you can use it to cultivate and you have already addressed your immediate challenge, which is access to food. So, donkeys could help people produce more food and if you had excess production, donkeys could help you transport it to the market where you could fetch a better price … and that becomes a very valuable role that donkeys can play in communities that have suffered some disastrous situation; to rebuild their lives, rebuild their livelihood.*

Donkeys play a critical role in recurrent and protracted crises, such as those produced by prolonged drought. Extreme weather events, including drought, fire, and flooding, are becoming increasingly commonplace because of climate change (Seneviratne et al., 2012). Donkeys are essential to the (changing) survival tactics of rural communities in East Africa's drylands, which are particularly vulnerable to the effects of climate change.

### Equids and humanitarian aid: providing access to food, water, and medicine

Equids are agile animals, able to traverse difficult terrain and to navigate steep and narrow paths that may be strewn with rubble after an attack or earthquake (TDS and Animal Nepal, 2016). For this reason, they have been used for decades to deliver humanitarian aid. During the First World War (1914–18), mules were employed by Allied troops to transport soldiers, equipment, and the sick and injured (Burden, 2014). In more recent times, equids have been used by local people to navigate conflict zones, such as Syria and the Occupied Palestinian Territories (Johnson, 2019). The Red Cross (2013) reports that in the early years of the Syrian conflict, horses were used by volunteers to deliver aid to affected families in hard to access areas or when there were fuel shortages, resulting in limited access to motorised vehicles.^10^ Research has also found that donkeys are increasingly being used as ‘ambulances’ in rural parts of Africa, where they transport the sick and injured to hospital (Ali et al., 2014).

Donkeys have reportedly played an essential role in delivering aid in times of extreme flooding. For instance, in parts of Kenya during the rainy season, roads can become impassable. Recent flooding in Iresaboru, Isiolo County, caused the displacement of families and donkeys were used to carry belongings, children, and the elderly to temporary shelters and other places of safety.^11^ Likewise, during the floods of 2014–15 in Turkana, northwest Kenya, communities were left entirely cut off from services and supplies. One interviewee (KI.4) recounted that:

*Many communities across the country relied on donkeys to relocate and deliver supplies because roads were badly damaged and vehicles could not pass … women who were going to seek maternal services in hospitals were carried by donkeys … those who deliver human health and veterinary services relied on donkeys to transport materials and equipment… . So, the donkey becomes a very handy animal in a crisis situation, in the sense that it can be used to deliver supplies, which communities badly need to survive a catastrophe.*

Another important example of the provision of humanitarian aid by donkeys comes from South Sudan, which has experienced a chronic emergency for the past 20 years or so as a result of civil war, characterised by many cycles of violent conflict. People have lived with the constant threat of having to flee, quickly and often for extended periods (UNHCR, 2019). When an attack is imminent, households must rapidly gather their possessions and disperse to safety. Donkeys are invaluable at this point. As one interviewee observed (KI.2):

*Donkeys were so critical that in some places it became mandatory that, as a household, out of the so many possessions you could have, the list [of items to take] could not be completed if you did not have a donkey … whatever household items they had would be loaded onto donkeys to be ferried away.*

In Northern Bahr el Ghazal State and Unity State between 2001 and 2018, donkeys were witnessed carrying the elderly, sick, and disabled, including those who were injured during the fighting; desperately trying to get away to avoid attacks by militia. One interviewee (KI.2) said: ‘All these people would be loaded on to donkeys to move them to safety. They would use donkeys for movement; they were like a mobility asset’. When people were finally able to return to their former settlements, donkeys were once again vital in helping people rebuild their lives and livelihoods. They helped to fetch water, firewood, and mudding/cladding so people could rebuild their homes.^12^ They also supported the re‐establishment of agricultural activities; ploughing fields and transporting goods to markets. Some donkey owners hired out their donkeys to others (including to those who had lost their own donkey during the conflict), generating an immediate source of income for themselves and their families while helping others to rebuild their lives.^13^

Not only do donkeys help to facilitate humanitarian aid, but they also provide an essential service in delivering veterinarian aid. In parts of northern Kenya and Somalia, where animal health services are limited owing to a lack of resources, conflict/security issues, rough terrain, and minimal transport/infrastructure (Catley, 1999), donkeys play a key part in the transportation of drugs/medicine and veterinary equipment, used by community animal health workers. They transport de‐wormers, antibiotics, and anti‐parasitic drugs for livestock owned by communities and herders in remote areas, helping to ensure their health and survival during difficult periods.^14^

These examples highlight the diversity of roles undertaken by donkeys and other working equids during times of crisis. Being such versatile animals, working equids can bolster a community's ability to respond to, cope with, and/or recover from the effects of a disaster or emergency. The following section discusses the importance of planning and making provisions for working equids in LMICs, especially poor animal‐dependent communities that may be particularly vulnerable to the effects of disasters or emergencies.

## Discussion

### Animal welfare supports human welfare: the need for adequate planning and provision for working equids in humanitarian crises

The field informants who we interviewed confirmed that the welfare of working equids is often poor in communities that rely on them heavily. In times of crisis, they can suffer from dehydration, exhaustion, and injury. They are put at immediate risk during armed conflicts, for instance, due to stray bullets or other weapons, and are sometimes loaded with explosive devices and sent to targeted areas, which can result in injury and death (VSF‐Suisse, 2020).^15^ Despite their contributions in times of crisis, the welfare of working equids is frequently overlooked. This can be because of myriad factors, including access to services, socioeconomic circumstances, and cultural norms and belief systems (Watson et al., 2020). Donkeys have a reputation for working tirelessly and without complaint, the ultimate ‘beast of burden’ (Bough, 2012), which can have implications for their welfare. As one field officer (KI.9) explained:

*In northern Somalia, people overuse the donkey … the idea is to ‘use the donkey well’ before it dies… . In times of drought, the pastoralists are always thinking about how they can ensure the other livestock survive, not the donkey. The donkey is the worker. The donkey is the one animal that is not given a chance to eat, a chance to rest. If the donkey is sick but can still work, they will still make the donkey work, but if it cannot work, they will leave it—and leave it to get eaten by the hyenas.*

Another field officer working in South Sudan and northern Kenya (KI.7) offered similar insights:

*It's a very bad situation. Through my work, I could see what critical role the donkey could play in supporting communities, households, owners. But the donkey was always the most neglected animal when it comes to welfare concerns.*

Perhaps partly due to their evolution, donkeys exhibit very subtle signs of pain or fear‐related distress, which are easily overlooked (TDS, 2017); welfare issues can be particularly acute among donkeys. To compound the issue, the matter of donkey health and welfare is also largely absent from the training and resources provided to veterinarians working for frontline services. One interviewee (KI.2) commented that:

*The manual used by frontline veterinarians and para‐veterinarians didn't refer to donkeys once. In training, they talked about goats, they talked about cattle and sheep and maybe chickens, but they never mentioned donkeys. You hardly had anyone who could handle a sick donkey. No one knew what problems they had clinically. No one knew how to approach a donkey when they needed treatment. No one knew about medications for donkeys. They might have general knowledge, like what antibiotic to inject, but out of 10 vets you could easily find zero who had ever injected a donkey to treat any condition or even wormed a donkey or even cleaned or trimmed a foot. So, there was a huge gap in knowledge among the frontline animal service workforce.*

Once again, this reflects the invisibility of donkeys in the wider context of international development, including the international response to a disaster by humanitarian and veterinarian organisations. As one key informant (KI.2) put it:

*donkeys clearly provide a benefit to communities, but it is a silent benefit: nobody documents it, nobody advocates on it, and it goes silent; unnoticed and taken for granted.*

As a result, rarely is adequate welfare provision made for donkeys and other working equids in times of crisis (Beirne and Kelty‐Huber, 2015; Pollock, 2018). Most international disaster relief agencies concentrate solely on the human aspect of emergency relief. As Sprayson (2006, p. 49) underlines: ‘such agencies do not take into account that the lives of many people throughout the world are inextricably intertwined with those of their livestock’. When in 2003, approximately 14,000 donkeys carried families displaced by war and a disaster triggered by natural hazards to the Abu Shouk refugee camp in Darfur, Sudan, only 2,300 donkeys were reported still to be alive 18 months later; the remaining donkeys (84 per cent) died from stress and a lack of feed and water (Pollock, 2018). Not only was this a huge loss of animal life, but also it was a huge loss for the owners, for whom the donkeys were the sole means of transport or of earning a living, as well as a lifeline to a future outside of the camp. However, these issues are rarely documented or attract media attention. Pollock (2018) found that there is almost no data on the number of working equids used to travel to or from refugee camps anywhere in the world. Little is known about their fate when they arrive, nor the tasks they are given or the conditions they face (Pollock, 2018).

For this reason, animals should be central to any analysis or assessment of vulnerability or resilience, with humanitarian, veterinarian, and welfare organisations working in close collaboration. Pollock (2018) suggests that refugee camps represent a great opportunity for humanitarian and veterinarian agencies to work together, since they often have similar needs and frequently use similar equipment. However, although camps are convenient, ultimately, they are less than ideal environments for their inhabitants and should be considered as a temporary solution until more humane alternatives are secured for both humans and animals alike (UNHCR, 2014). Nevertheless, collaborative efforts will undoubtedly ‘make a difference for the long‐term benefit of displaced people and their animals’ (Pollock, 2018, p. 8). Moreover, as White (2020, p. 2) puts it, ‘if humanitarian assistance does not extend to the animals, the people's experience of displacement will worsen’. This serves as a reminder of the close link between animal and human welfare, chiming with recent calls to adopt a ‘One Welfare’ approach^16^ in relation to international development (WHW and TDS, 2020).

### Shared vulnerability and resilience in the context of humanitarian crises

The lives of humans and animals are intimately entangled, often especially so in times of crisis (Beirne and Kelty‐Huber, 2015; Johnson, 2019). As this paper has demonstrated, equids can play a pivotal role in the response and/or recovery efforts of many LMIC communities affected by a crisis. They can provide a source of income and/or livelihood generation during times of drought and a means of transport to safety for those displaced by armed conflict. They can provide access to food, water, and medicine, inter alia, during disasters triggered by natural hazards and extreme weather events. They can enable the survival of other important livestock species during times of drought. They can provide a cultural tie; a sense of familiarity among those displaced from their homes. Yet, despite their contributions and essential relations, working animals (particularly equids) remain largely invisible in the academic literature on disasters and emergencies (White, 2020). Likewise, the vast majority of work on resilience vis‐à‐vis disasters and emergencies does not fully explore the centrality of working animals in LMICs, by foregrounding their place and role in response and/or recovery endeavours. This means that international development initiatives and policies can overlook the complex ways that resilience and vulnerability are shared and co‐produced alongside nonhuman animals.

Working equids play diverse socioeconomic roles and contribute to economic development and social welfare by providing means of poverty reduction and income security, enabling the continuity of agricultural supply chains and generating access to nutrition and water—all of which contributes to the creation and maintenance of sustainable livelihoods (The Brooke, 2019). Recently, specialists and practitioners have begun to emphasise the importance of prioritising the long‐term health and welfare of working animals in humanitarian crises (WHW and TDS, 2020). International agencies, when they do consider the welfare of working animals, generally provide reactive support in the *aftermath* of a disaster or crisis, that is, in the shape of emergency veterinary aid and/or feed. But scholars have argued that reactionary work in the form of relief is not enough and that more needs to be done at the planning and mitigation stage (Sprayson, 2006). Instead, scholars suggest that more emphasis needs to be put on strategic animal health policies and long‐term investment (Sprayson, 2006; Alshawawreh, 2018). According to Sprayson (2006), preparing for disasters and building resilience within animal‐dependent communities involves the creation of adequate housing for the animals, good access to forage and back‐up feed options (in case of an emergency), regular worming, husbandry, and vaccination programmes. In many cases, though, social and political issues may hinder and complicate these well‐intentioned efforts. There may be underlying social and structural inequalities that mean that some people can access services/support while others cannot (Bankoff, Hilhorst, and Frerks, 2004; Bryant, 2015).

There are always multiple factors that result in unequal exposure to the impacts of disasters, including class, race, gender, and wealth. Working equids can help to mitigate the impacts on their owners, but equid ownership alone does not resolve underlying structural and social inequality. Resilience‐building and disaster mitigation for vulnerable communities (including animal‐dependent communities) requires, therefore, a holistic package of long‐term service provision, including access to education, resources, health services, work opportunities, rights, and security.

## Conclusion

We have outlined in this paper the versatility of working equids and the variety of functions they take on in crisis situations. Dependency on working equids is part of the social and political landscape of many poor and marginalised communities in LMICs. Without working equids, the vulnerabilities of many people would be even more stark or exposed. Yet, the contributions of working equids often go unseen. The vast majority of resilience research has overlooked the parts that they play in disasters and emergencies in LMICs, including the extent to which they alleviate the impacts on vulnerable communities. Likewise, working equids are largely invisible in international policy and planning; they are not considered along with other livestock species.

Persuading policymakers to recognise working equids (and providing the budgets to support them) in challenging and complex emergencies will require strong evidence of their importance in aiding and supporting the resilience of communities in LMICs, particularly so given emerging climate‐related crises. It will be the responsibility of researchers and practitioners to evidence the contributions of working equids in these vulnerable communities, something which is currently lacking but vital to advancing the place of working equids on global policy and humanitarian agendas. This evidence may be hidden: for example, while dealing with initial responses to crises, there may be other more pressing priorities, and so documentation on the contributions of working equids may be lost or overlooked. It is imperative, therefore, that efforts uncover this information and distribute the findings to all stakeholders.

For this information to be applied in practice, humanitarian practitioners should broaden their focus to view humans and animals as interlinked in times of crisis in LMIC communities where animals are relied on heavily. To aid recovery, communities in LMICs need human and animal welfare practitioners to collaborate actively to ensure that the needs of working animals are met and that their owners are given access to the resources required to sustain health, well‐being, and ultimately recovery. To do so requires the adoption of long‐term investment in animal health and the policies needed to support it, alongside the holistic provision of services to their owners: a truly collaborative effort to address a complex and urgent problem.

## Acknowledgements

We would like to thank all of the study participants for giving up their time to speak with us. We are extremely grateful to The Donkey Sanctuary and its supporters for funding this research and for furthering donkey and mule welfare worldwide. Finally, the authors declare no conflict of interest.

## Data availability statement

The data that support the findings of this study are available from the corresponding author upon reasonable request.^17^

## References

[disa12501-bib-0001] Admassu, B. and Y. Shiferaw (2011) Donkeys, Horses and Mules – Their Contribution to People's Livelihoods in Ethiopia. The Brooke, Addis Ababa.

[disa12501-bib-0002] Ali, M. , A. Baber , T. Hussain , F. Awan , and A. Nadeem (2014) ‘The contribution of donkeys to human health’. Equine Veterinary Journal. 46(6). pp. 766–767. 25319161 10.1111/evj.12337

[disa12501-bib-0003] Alshawawreh, L. (2018) ‘Sheltering animals in refugee camps’. Forced Migration Review. 58 (June). pp. 76–78.

[disa12501-bib-0004] Angeloni, G. and J. Carr (2018) ‘Animal and human health in the Sahrawi refugee camps’. Forced Migration Review. 58 (June). pp. 80–82.

[disa12501-bib-0005] Arriaga-Jordan, C.M. , A.M. Pedraza-Fuentes , L.G. Velazquez-Beltran , E.G. Nava-Bernal , and M.C. Chavez-Mejıa (2005) ‘Economic contribution of draught animals to Mazahua smallholder Campesino farming systems in the highlands of central Mexico’. Tropical Animal Health and Production. 37(7). pp. 589–597. 16450864 10.1007/s11250-005-4177-3

[disa12501-bib-0006] ASFG (African Smallholders Farmers Group) (2010) Africa's Smallholder Farmers: Approaches that Work for Viable Livelihoods. https://reliefweb.int/sites/reliefweb.int/files/resources/B81F819A0C4ADEB385257760005E85F2-pa-asfg-africa-smallholder-farmers-jul2010.pdf (last accessed on 10 June 2022).

[disa12501-bib-0007] Asiimwe, R. , J.H. Ainembabazi , and A. Egeru (2020) ‘The role of camel production on household resilience to droughts in pastoral and agro-pastoral households in Uganda’. Pastoralism. 10. Article number: 5. 10.1186/s13570-020-0160-x.

[disa12501-bib-0008] Avornyo, F. , G. Teye , D. Bukari , and S. Salifu (2015) ‘Contribution of donkeys to household food security: a case study in the Bawku municipality of the Upper East Region of Ghana’. Ghana Journal of Science, Technology and Development. 3(1). pp. 15–24.

[disa12501-bib-0009] Bankoff, G. , D. Hilhorst , and G. Frerks (2004) Mapping Vulnerability: Disasters, Development and People. First edition. Earthscan, Abingdon.

[disa12501-bib-0010] Beirne, P. and C. Kelty-Huber (2015) ‘Animals and forced migration’. Forced Migration Review. 49 (May). pp. 97–98.

[disa12501-bib-0011] Béné, C. , A. Newsham , M. Davies , M. Ulrichs , and R. Godfrey-Wood (2014) ‘Review article: resilience, poverty and development’. Journal of International Development. 26(5). pp. 598–623.

[disa12501-bib-0012] Biernacki, P. and D. Waldorf (1981) ‘Snowball sampling: problems and techniques of chain 899 referral sampling’. Sociological Methods and Research. 10(2). pp. 141–163.

[disa12501-bib-0013] Bough, J. (2012) Donkey. Reaktion Books, London.

[disa12501-bib-0014] Brown, K. (2016) Resilience, Development and Global Change. Routledge, New York, NY.

[disa12501-bib-0015] Bryant, R. (2015) The International Handbook of Political Ecology. Edward Elgar Publishing, Cheltenham.

[disa12501-bib-0016] Burden, F. (2014) ‘Of mules and men: challenging relationships in WW1’. Presentation to the ‘War Horses of the World’ conference, Department of History, School of History, Religions and Philosophies, SOAS University of London, London, United Kingdom, 3 May 2014. https://www.soas.ac.uk/history/conferences/war-horses-conference-2014/ (last accessed on 10 June 2022)

[disa12501-bib-0017] Cannon, T. and D. Müller-Mahn (2010) ‘Vulnerability, resilience and development discourses in context of climate change’. Natural Hazards. 55(3). pp. 621–635.

[disa12501-bib-0018] Catley, A. (1999) Community-based Animal Health Care in Somali Areas of Africa: A Review. Prepared for the Participatory Community-based Vaccination and Animal Health Project of the Organisation of African Unity and the Interafrican Bureau for Animal Resources. https://www.alnap.org/system/files/content/resource/files/main/somalicahw.pdf (last accessed on 10 June 2022)

[disa12501-bib-0019] CFS (Committee on World Food Security) (2016) The Role of Livestock in Sustainable Agriculture: Delivering for People, Animals and Planet. Side event, 43rd Session (CFS 43), FAO Headquarters, Rome, Italy, 17–21 October 2016.

[disa12501-bib-0020] Christian Aid (2012) Thriving, Resilient Livelihoods: Christian Aid's Approach. Briefing. October. Christian Aid, London.

[disa12501-bib-0021] Claus, C. et al. (2015) ‘Disaster, degradation, dystopia’. In R. Bryant (ed.) The International Handbook of Political Ecology. Edward Elgar Publishing, Cheltenham. pp. 291–304.

[disa12501-bib-0022] Cross, R. (2015) ‘Nepal earthquake: a disaster that shows quakes don't kill people, buildings do’. The Guardian. 30 April. https://www.theguardian.com/cities/2015/apr/30/nepal-earthquake-disaster-building-collapse-resilience-kathmandu (last accessed on 10 June 2022).

[disa12501-bib-0023] DFID (Department for International Development) (2011) Defining Disaster Resilience: A DFID Approach Paper. DFID, London.

[disa12501-bib-0024] Echeverri, A. , D. Karp , R. Naidoo , J. Zhao , and K. Chana (2018) ‘Approaching human–animal relationships from multiple angles: a synthetic perspective’. Biological Conservation. 224 (August). pp. 50–62.

[disa12501-bib-0025] Fabinyi, M. , L. Evans , and S.J. Foale (2014) ‘Social-ecological systems, social diversity, and power: insights from anthropology and political ecology’. Ecology and Society. 19(4). Article number: 28. 10.5751/ES-07029-190428.

[disa12501-bib-0026] FAO (Food and Agriculture Organization of the United Nations) (2011) FAO Statistical Yearbook 2011. FAO, Rome.

[disa12501-bib-0027] FAO (2016) Livestock in Protracted Crises: The Importance of Livestock for Resilience-building and Food Security of Crisis-affected Populations. Guidance Note. FAO, Rome.

[disa12501-bib-0028] Field, C.B. et al. (2014) Climate Change 2014 – Impacts, Adaptation and Vulnerability: Part A: Global and Sectoral Aspects. Working Group II Contribution to the Fifth Assessment Report of the Intergovernmental Panel on Climate Change. Cambridge University Press, New York, NY.

[disa12501-bib-0029] Gaillard, J.C. (2010) ‘Vulnerability, capacity and resilience: perspectives for climate and development policy’. Journal of International Development. 22(2). pp. 218–232.

[disa12501-bib-0030] Ghislain, S. (2019) Animal Welfare, Trade and Sustainable Development Goals. Policy Brief. Eurogroup for Animals, Brussels.

[disa12501-bib-0031] Grove, K. (2018) Resilience. Routledge, New York, NY.

[disa12501-bib-0032] He, L. , J.C. Aitchison , K. Hussey , Y. Wei , and A. Lo (2018) ‘Accumulation of vulnerabilities in the aftermath of the 2015 Nepal earthquake: household displacement, livelihood changes and recovery challenges’. International Journal of Disaster Risk Reduction. 31 (October). pp. 68–75.

[disa12501-bib-0033] Herz, M. (2012) From Camp to City: Refugee Camps of the Western Sahara. Lars Muller Publishers, Baden.

[disa12501-bib-0034] Hoots, C. (2018) ‘The role of livestock in refugee–host community relations’. Forced Migration Review. 58 (June). pp. 71–74.

[disa12501-bib-0035] Humanitarian Coalition (2020) ‘What is a humanitarian emergency?’. Website. https://www.humanitariancoalition.ca/info-portal/factsheets/what-is-a-humanitarian-crisis (last accessed on 10 June 2022).

[disa12501-bib-0036] Hussein, M. (2013) ‘Resilience: meaningless jargon or development solution?‘. The Guardian. 5 March. https://www.theguardian.com/global-development-professionals-network/2013/mar/05/resilience-development-buzzwords (last accessed on 10 June 2022).

[disa12501-bib-0037] Izraely, H. , I. Choshniak , C.E. Stevens , and A. Shkolnik (1989) ‘The donkey: coping with low quality feed’. Asian-Australasian Journal of Animal Sciences. 2(3). pp. 289–291.

[disa12501-bib-0038] Johnson, P. (2019) Companions in Conflict: Animals in Occupied Palestine. Melville House Publishing, London.

[disa12501-bib-0039] Kimura, A. (2015) ‘Understanding Fukushima: nuclear impacts, risk perceptions and organic farming in a feminist political ecology perspective’. In R. Bryant (ed.) The International Handbook of Political Ecology. Edward Elgar Publishing, Cheltenham. pp. 260–273.

[disa12501-bib-0040] Klein, R.J.T. and J.B. Smith (2003) ‘Enhancing the capacity of developing countries to adapt to climate change: a policy-relevant research agenda’. In J.B. Smith , R.J.T. Klein , and S. Huq (eds.) Climate Change, Adaptive Capacity and Development. Imperial College Press, London. pp 317–334.

[disa12501-bib-0041] Klingel, H. (1990) ‘Horses’. In B. Grzimek (ed.) Grzimek's Encyclopedia of Mammals. McGraw-Hill Publishing Company, New York, NY. pp. 557–597.

[disa12501-bib-0042] Leach, M. (2008) Re-framing Resilience: A Symposium Report. STEPS Working Paper 13. STEPS (Social, Technological and Environmental Pathways to Sustainability) Centre, University of Sussex, Brighton.

[disa12501-bib-0043] LEGS Project (2014) Livestock Emergency Guidelines and Standards (LEGS). Handbook. Second edition. Practical Action Publishing, Rugby.

[disa12501-bib-0044] LEGS Project (2018) Supporting Livelihoods and Livestock during Drought in Pastoralist Areas: The Livelihoods and Nutritional Impacts of LEGS Interventions. LEGS Briefing Paper. March. LEGS Project, Great Holland.

[disa12501-bib-0045] Lindenmayer, J. (2008) ‘Animals as key promoters of human resilience’. African Health Sciences. 8(S1). Article number: 46. PMID: 21448373; PMCID: PMC3060723. PMC3060723 21448373

[disa12501-bib-0046] MacKinnon, D. and K.D. Derickson (2012) ‘From resilience to resourcefulness – a critique of resilience policy and activism’. Progress in Human Geography. 37(2). pp. 253–270.

[disa12501-bib-0047] Martin-Breen, P. and J.M. Anderies (2011) Resilience: A Literature Review. Bellagio Initiative, Institute of Development Studies, Brighton.

[disa12501-bib-0048] Muzzini, E. and G. Aparicio (2013) Urban Growth and Spatial Transition in Nepal: An Initial Assessment. Directions in Development. World Bank, Washington, DC.

[disa12501-bib-0049] Mwasame, D.B. (2020) Analysis of the Socio-economic Contribution of Donkey Ownership and Use to Household Livelihoods in Kiambu County. A thesis submitted in partial fulfilment for the award of the degree of Master of Science in agricultural and applied economics, Department of Agricultural Economics, Faculty of Agriculture, University of Nairobi, Kenya. A56/83706/2015. http://erepository.uonbi.ac.ke/bitstream/handle/11295/153130/Mwasame_Analysis%20of%20the%20Socio-economic%20contribution%20of%20donkey%20ownership%20and%20use%20to%20Household%20Livelihoods%20in%20Kiambu%20County%2C%20Kenya.pdf?sequence=1&isAllowed=y (last accessed on 10 June 2022).

[disa12501-bib-0050] Nazrul Islam, S. and J. Winkel (2017) Climate Change and Social Inequality. DESA Working Paper No. 152. ST/ESA/2017/DWP/152. October. UN Department of Economic and Social Affairs, New York, NY.

[disa12501-bib-0051] OSOCC (On-Site Operations Coordination Centre) (2015) Situation Analysis: Nepal Earthquake. 15 May. https://reliefweb.int/sites/reliefweb.int/files/resources/150515_nepal_situation_analysis_osocc_assessmente_cell_-_final_final.pdf (last accessed on 10 June 2022).

[disa12501-bib-0052] Owczarczak-Garstecka, S. (2018) ‘Understanding risk in human–animal interactions’. Forced Migration Review. 58 (June). pp. 78–80.

[disa12501-bib-0053] Perrings, C. (2006) ‘Resilience and sustainable development’. Environment and Development Economics. 11(4). pp. 417–427.

[disa12501-bib-0054] Perry, B. (2017) ‘We must tie equine welfare to international development’. Veterinary Record. 181(22). pp. 600–601. 29192048 10.1136/vr.j5561

[disa12501-bib-0055] Pollock, P. (2018) ‘Working equids in refugee camps’. Forced Migration Review. 58 (June). pp. 7–8.

[disa12501-bib-0056] Porter, L. and S. Davoudi (2012) ‘The politics of resilience for planning: a cautionary note’. Planning Theory and Practice. 13(2). pp. 329–333.

[disa12501-bib-0057] Pritchard, J. (2014) ‘What role do working equids play in human livelihoods and how well is this currently recognised?’. In J. Wade (ed.) Proceedings of the 7th International Colloquium on Working Equids. Royal Holloway, University of London, London, United Kingdom, 1–3 July 2014. https://docplayer.net/56704640-Proceedings-of-the-7-th-international-colloquium-on-working-equids.html (last accessed on 10 June 2022). pp. 2–6.

[disa12501-bib-0058] Rahman, S. and K. Reed (2014) ‘The management and welfare of working animals: identifying problems, seeking solutions and anticipating the future’. Revue Scientifique et Technique de l'OIE. 33(1). pp. 197–202 10.20506/rst.33.1.2272 25000792

[disa12501-bib-0059] Rawlence, B. (2016) City of Thorns: Nine Lives in the World's Largest Refugee Camp. Picador, New York, NY.

[disa12501-bib-0060] Red Cross (2013) ‘Photo of the day: innovative way to deliver aid in Syria’. Website. 8 October. https://www.redcross.ca/blog/2013/10/photo-of-the-day-innovative-way-to-deliver-aid-in (last accessed on 10 June 2022).

[disa12501-bib-0061] ReliefWeb (2008) Glossary of Humanitarian Terms. August. https://reliefweb.int/report/world/reliefweb-glossary-humanitarian-terms-enko (last accessed on 13 June 2022).

[disa12501-bib-0062] Seneviratne, S.I. et al. (2012) ‘Changes in climate extremes and their impacts on the natural physical environment’. In C.B. Field et al. (eds.) Managing the Risks of Extreme Events and Disasters to Advance Climate Change Adaptation: A Special Report of Working Groups I and II of the Intergovernmental Panel on Climate Change (IPCC). Cambridge University Press, New York, NY. pp. 109–230.

[disa12501-bib-0063] Smith, D.G. and R.A. Pearson (2005) ‘A review of the factors affecting the survival of donkeys in semi-arid regions of Sub-Saharan Africa’. Tropical Animal Health Production. 37 (January). pp. 1–19. 10.1007/s11250-005-9002-5 16335068

[disa12501-bib-0064] Sprayson, T. (2006) ‘Taking the lead: veterinary intervention in disaster relief’. InPractice. 28(1). pp. 48–51.

[disa12501-bib-0065] Starkey, P. and M. Starkey (2004) ‘Regional and world trends in donkey populations’. In P. Starkey and D. Fielding (eds.) Donkeys, People and Development: A Resource Book of the Animal Traction Network for Eastern and Southern Africa (ATNESA). ACP-EU Technical Centre for Agricultural and Rural Cooperation (CTA), Wageningen, The Netherlands. pp. 33–44.

[disa12501-bib-0066] Stringer, A. (2014) ‘Improving animal health for poverty alleviation and sustainable livelihoods’. Veterinary Record. 175(21). pp. 526–529. 25431381 10.1136/vr.g6281

[disa12501-bib-0067] TDS (The Donkey Sanctuary) (2017) Understanding Donkey Behaviour: Factors that Influence a Donkey's Behaviour. TDS, Sidmouth.

[disa12501-bib-0068] TDS and Animal Nepal (2016) Supporting Mountain Mules: ‘A Survey on Working Mules of Gorkha’. Animal Nepal, Kathmandu.

[disa12501-bib-0069] The Brooke (2019) Invisible Livestock: Benefits, Threats and Solutions. https://www.thebrooke.org/sites/default/files/Advocacy-and-policy/Invisible-livestock-2019.pdf (last accessed on 10 June 2022).

[disa12501-bib-0070] The Brooke (2016) ‘UN recognises role of working animals in food security’. 19 October. https://www.thebrooke.org/news/un-recognises-working-animals-role (last accessed on 10 June 2022).

[disa12501-bib-0071] Tierney, K. (2014) The Social Roots of Risk: Producing Disasters, Promoting Resilience. Stanford University Press, Palo Alto, CA.

[disa12501-bib-0072] UN (United Nations) (1987) Our Common Future. Report of the World Commission on Environment and Development (The Brundtland Report). UN, Oslo.

[disa12501-bib-0073] UN (2005a) The Transition from Relief to Development: Report of the Secretary-General. UN General Assembly Economic and Social Council. A/60/89–E/2005/79. 23 June. https://reliefweb.int/sites/reliefweb.int/files/resources/71EBD3A62CCA05E48525703E007305BB-unga-gen2-23jun.pdf (last accessed on 10 June 2022).

[disa12501-bib-0074] UN (2005b) Hyogo Framework for Action 2005–2015: Building the Resilience of Nations and Communities to Disasters. World Conference on Disaster Reduction, Kobe, Hyogo, Japan, 18–22 January 2005. https://www.unisdr.org/2005/wcdr/intergover/official-doc/L-docs/Hyogo-framework-for-action-english.pdf (last accessed on 10 June 2022).

[disa12501-bib-0075] UN (2009) 2009 UNISDR Terminology on Disaster Risk Reduction. United Nations International Strategy for Disaster Risk Reduction. https://www.unisdr.org/files/7817_UNISDRTerminologyEnglish.pdf (last accessed on 10 June 2022).

[disa12501-bib-0076] UN (2015a) Sendai Framework for Disaster Risk Reduction. https://sustainabledevelopment.un.org/content/documents/2157sendaiframeworkfordrren.pdf (last accessed on 10 June 2022).

[disa12501-bib-0077] UN (2015b) Transforming our World: 2030 Agenda for Sustainable Development. A/RES/70/1. https://sustainabledevelopment.un.org/content/documents/21252030%20Agenda%20for%20Sustainable%20Development%20web.pdf (last accessed on 10 June 2022).

[disa12501-bib-0078] UN (2020) ‘Emergency and disaster management’. Office for Outer Space Affairs, UN-SPIDER Knowledge Portal. https://un-spider.org/risks-and-disasters/emergency-and-disaster-management (last accessed on 10 June 2022).

[disa12501-bib-0079] UNDP (United Nations Development Programme) (2016) Sustainable Urbanization Strategy: UNDP's Support to Sustainable, Inclusive and Resilient Cities in the Developing World. UNDP, New York, NY.

[disa12501-bib-0080] UNESCO (United Nations Educational, Scientific and Cultural Organization) (2015) ‘UNESCO to assess the impact on Nepal's cultural heritage of the devastating earthquake’. Website. 27 April. https://whc.unesco.org/en/news/1268 (last accessed on 10 June 2022).

[disa12501-bib-0081] UNHCR (United Nations Refugee Agency) (2014) UNHCR Policy on Alternatives to Camps. https://www.unhcr.org/uk/protection/statelessness/5422b8f09/unhcr-policy-alternatives-camps.html (last accessed on 13 June 2022)

[disa12501-bib-0082] UNHCR (2016) Integration — A Fundamental Component in Supporting Diverse Societies. https://www.unhcr.org/56a9decf5.pdf (last accessed on 10 June 2022).

[disa12501-bib-0083] UNHCR (2019) Global Trends: Forced Displacement in 2019. Statistics and Demographics Section, UNHCR Global Data Service, Copenhagen.

[disa12501-bib-0084] Valette, D. (2015) Invisible Workers: The Economic Contributions of Working Donkeys, Horses and Mules to Livelihoods. The Brooke, London.

[disa12501-bib-0085] van Dijk, L. and J. Pritchard (2014) ‘Working animals’ potential to reduce vulnerability to the effects of climate change'. In J. Wade (ed.) How Do We Demonstrate the Importance of Working Equid Welfare To Human Livelihoods?: The Proceedings of the 7th International Colloquium on Working Equids. Royal Holloway, University of London, United Kingdom, 1–3 July 2014. World Horse Welfare, Norwich. pp. 51–52.

[disa12501-bib-0086] VSF (Vétérinaires Sans Frontières) International (2018) From Emergency to Development. Building Resilience through Livestock-based Interventions. VSF International Policy Paper No. 4. February. VSF International, Brussels.

[disa12501-bib-0087] Watson, E.E. , H.H. Kochore , and B.H. Dabasso (2016) ‘Camels and climate resilience: adaptation in northern Kenya’. Human Ecology. 44(6). pp. 701–713. 10.1007/s10745-016-9858-1 PMC5143358 28018023

[disa12501-bib-0088] Watson, T.L. et al. (2020) ‘Cultural “blind spots,” social influence and the welfare of working donkeys in brick kilns in northern India’. Frontiers in Veterinary Science. 7(214). 10.3389/fvets.2020.00214. PMC7201042 32411736

[disa12501-bib-0089] Welsh, M. (2014) ‘Resilience and responsibility: governing uncertainty in a complex world’. The Geographic Journal. 180(1). pp. 15–26

[disa12501-bib-0090] WFP (World Food Programme) (2019) R4 Rural Resilience Initiative. Factsheet. 1 October. https://www.wfp.org/publications/r4-rural-resilience-initiative (last accessed on 10 June 2022).

[disa12501-bib-0091] White, B. (2018) ‘Humans and animals in refugee camps’. Forced Migration Review. 58 (June). pp. 2–3.

[disa12501-bib-0092] White, B. (2020) ‘Animals, people and places in displacement’. In P. Adey et al. (eds.) The Handbook of Displacement. Palgrave Macmillan, London. pp. 549–568.

[disa12501-bib-0093] WHW (World Horse Welfare) and TDS (The Donkey Sanctuary) (2017) Sustainable Development Goals: How the Welfare of Working Equids Delivers for Development. Policy Briefing. https://www.thedonkeysanctuary.org.uk/sites/uk/files/2018-03/sustainable-development-goals.pdf (last accessed on 10 June 2022).

[disa12501-bib-0094] WHW and TDS (2020) One Health and One World: Working Animals to Help Achieve a Safe and Sustainable World. https://sustainabledevelopment.un.org/content/documents/26709DonkeyClimate_Change_Public_Health_Working_Animals.pdf (last accessed on 10 June 2022).

[disa12501-bib-0095] Wisner, B. , P. Blaikie , T. Cannon , and I. Davis (2004) At Risk: Natural Hazards, People's Vulnerability and Disasters. Second edition. Routledge, London.

[disa12501-bib-0096] WRI (World Resources Institute) (2008) World Resources 2008: Roots of Resilience. Growing the Wealth of the Poor. WRI, Washington, DC.

[disa12501-bib-0097] Yin, R. (2013) Case Study Research: Design and Methods. Fifth edition. Sage Publications, Newbury Park, CA.

